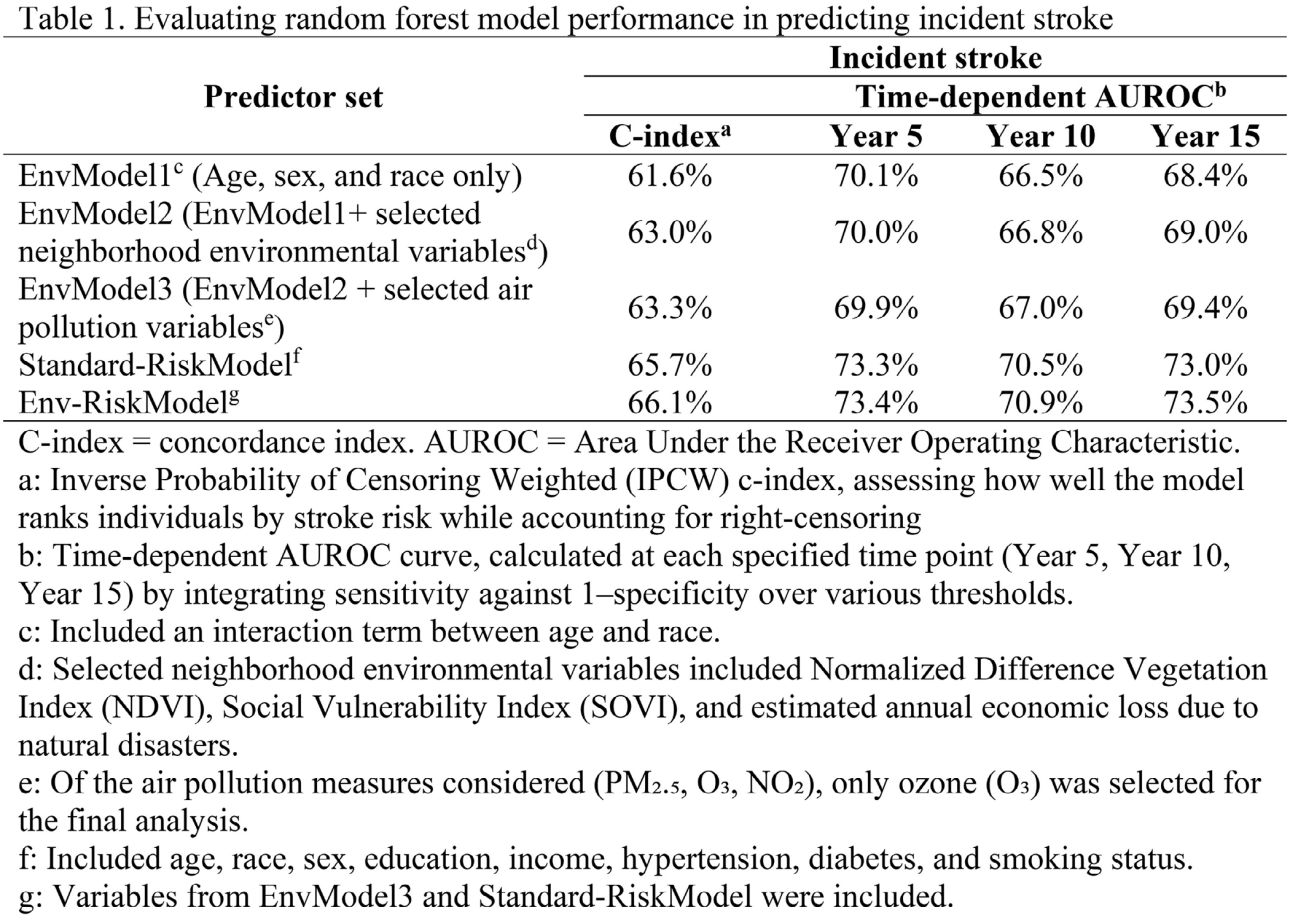# Environmental Contributions to Stroke Prediction: A Multi‐Dimensional Exposome Index

**DOI:** 10.1002/alz70860_105669

**Published:** 2025-12-23

**Authors:** Yi‐Han Hu, Harris Jamal, Xinlei Deng, Emily J. Werder, Virginia J Howard, Suzanne E Judd, Leslie A. McClure, Dale P. Sandler, Lenore J J. Launer

**Affiliations:** ^1^ Laboratory of Epidemiology and Population Sciences, National Institute on Aging, Baltimore, MD, USA; ^2^ Epidemiology Branch, National Institute of Environmental Health Sciences, Durham, NC, USA; ^3^ School of Public Health, University of Alabama at Birmingham, Birmingham, AL, USA; ^4^ College for Public Health and Social Justice, Saint Louis University, St. Louis, MO, USA

## Abstract

**Background:**

Stroke is a major risk factor for vascular dementia. Traditional stroke prediction models rely on standard individual‐level risk factors, but environmental exposures may also influence cerebrovascular health. Here, we use machine learning models to develop an environmental risk prediction model (Env‐RiskModel) and compare it to a prediction model with standard risk factors for stroke (Standard‐RiskModel).

**Method:**

Data are from the REasons for Geographic and Racial Differences in Stroke (REGARDS) study (2003–2007, *n* = 24,251; average age 64.6 years, 53.5% female, 42.2% Black). Adjudicated incident strokes were identified through September 2022. Environmental exposure measures 1‐yr prior to enrollment were linked to geocoded residential locations. Five random survival forest models were developed using an 80/20 train‐test split. Environmental variables were selected with staged Boruta models that identified key predictors from all environmental candidate variables; bootstrapping Area Under the Receiver Operating Characteristic (AUROC) confirmed their contribution beyond basic demographics. EnvModel1 included age, sex, and race. EnvModel2 added neighborhood characteristics (greenspace, social vulnerability, and disaster risk) to EnvModel1. EnvModel3 further added ambient air pollution (Ozone) to EnvModel2. The ‘Standard‐RiskModel’ included age, sex, race, education, income, hypertension, diabetes and smoking. The ‘Env‐RiskModel’ combined all variables from EnvModel3 and the Standard‐RiskModel. Comparisons of model performance were assessed by C‐index and time‐dependent AUROC.

**Results:**

Over a median 12.1‐years of follow‐up, 6.0% of participants experienced an incident stroke. EnvModel1 yielded a C‐index of 61.6%. Adding neighborhood variables in EnvModel2 increased the C‐index to 63.0%. EnvModel3 further incorporated Ozone, resulting in a slight increase in performance to 63.3%. Env‐RiskModel reached a C‐index of 66.1%, showing no meaningful improvement over Standard‐RiskModel (65.7%). Time‐dependent AUROCs also showed minimal differences between Standard‐RiskModel and Env‐RiskModel.

**Conclusions:**

Although neighborhood and air pollution factors did not significantly improve stroke risk prediction beyond individual‐level risk factors (Standard‐RiskModel vs Env‐RiskModel), combining environmental exposures with basic demographics (EnvModel3) performed nearly as well as the Standard‐RiskModel, suggesting environmental factors may still inform stroke prediction when disease‐behavior data are limited or unavailable. Further research is needed to evaluate the contribution to stroke prediction of additional environmental variables.